# Clustered repetitive transcranial magnetic stimulation for the prevention of depressive relapse/recurrence: a randomized controlled trial

**DOI:** 10.1038/s41398-017-0001-x

**Published:** 2017-12-18

**Authors:** Hua-Ning Wang, Xiao-Xia Wang, Rui-Guo Zhang, Ying Wang, Min Cai, Ya-Hong Zhang, Run-Zhu Sun, Li Guo, Yu-Ting Qiao, Jun-Chang Liu, Hong He, Zhong-Heng Wang, Yu-Chen Wan, Qing-Rong Tan, Zhang-Jin Zhang

**Affiliations:** 1 Department of Psychiatry, Xijing Hospital, The Fourth Military Medical University, 17 Changle Road, Xi’an, Shaanxi 710021 China; 2 School of Chinese Medicine, LKS Faculty of Medicine, The University of Hong Kong, 10 Sassoon Road, Pokfulam, Hong Kong, China

## Abstract

Repetitive transcranial magnetic stimulation (rTMS) may have the potential to prevent depressive relapse. This assessor-blinded, randomized controlled study was designed to evaluate the efficacy and safety of rTMS as a mono- and combination therapy in the prevention of depressive relapse/recurrence. A total of 281 depressed patients who had achieved stable full or partial remission on a 6-month antidepressant (ADP) run-in treatment were randomly assigned to an rTMS (*n* = 91), ADP (*n* = 108), or combined (rTMS + ADP, *n* = 82) treatment group for 12 months. Monthly clustered rTMS was conducted in 5–10 sessions over a 3–5-day period. Maintenance outcomes were assessed using time to relapse/recurrence and relapse/recurrence rate. Overall, 71.2% (200/281) of the participants completed the treatment per the protocol. rTMS + ADP and rTMS significantly reduced the risk of relapse/recurrence compared with ADP (*P* = 0.000), with hazard ratios of 0.297 and 0.466, respectively. Both rTMS-containing regimens produced significantly lower relapse/recurrence rates than ADP (15.9% and 24.2% vs. 44.4%, *P* < 0.001). In the relapsed/recurrent subgroup, first-episode depressed, rTMS-treated patients had a markedly lower relapse/recurrence rate than ADP-treated patients. Five patients on the ADP-containing regimens, but none on rTMS alone, developed acute mania. The rTMS-containing regimens had considerably more certain side effects than did the ADP group. We concluded that TMS, whether as a mono- or additional therapy, is superior to antidepressants in preventing depressive relapse/recurrence, particularly in first-episode depressed patients. The treatment does not increase the risk of manic switch, but may increase the risk of certain side effects.

## Introduction

Major depressive disorder (MDD) is often characterized by a relapsing or recurring course^1^. Although antidepressant maintenance therapy is the mainstay in the prevention of depressive relapse and recurrence, many patients still experience multiple depressive episodes over their life time^2,3^. Furthermore, the high level of non-adherence to long-term pharmacological maintenance treatment is an important risk factor for depressive relapse/recurrence^4^. Non-pharmacological interventions are, therefore, highly desirable.

As a noninvasive brain stimulation therapy, repetitive transcranial magnetic stimulation (rTMS) has been widely used to treat neurological and psychiatric disorders since 1985^5^. The U.S. Food and Drug Administration approved rTMS as a treatment for treatment-resistant MDD in 2008^6^. The acute and short-term antidepressant efficacy of rTMS has been well established in both depressed patients^7,8^ and experimental animal models^9^. The potential of rTMS for preventing depressive relapse and recurrence has also been demonstrated in four pilot controlled trials^10–13^ and eight case reports^14–21^. These studies encouraged us to conduct a controlled cohort study to acquire definitive evidence for the preventive efficacy of rTMS.

We hypothesized that rTMS could be an effective long-term maintenance treatment in preventing depressive recurrence. To test this hypothesis, we designed an assessor-blind, randomized controlled trial to evaluate the efficacy and safety profile of rTMS as a mono- and combination therapy compared with antidepressants alone as a 12-month maintenance treatment in patients who had achieved partial or full remission from antidepressant treatment of the current depressive episode.

## Methods

### Setting and participants

This assessor-blind, randomized controlled trial was conducted in the Department of Psychiatry of Xijing Hospital of Fourth Military University at Xi’an, China between January 2013 and May 2015. The study protocol was approved by the Medical Ethical Committee of Xijing Hospital and was registered at www.clinicaltrials.gov (NCT01516931)^22^. All participants gave voluntary, written, informed consent before entering the study.

In- and out-patients of either gender, aged 18–65, and currently experiencing a moderate or severe depressive episode according to the International Classification of Diseases (10th version) (ICD-10)^23^, as evidenced by a score of ≥18 on the 17-item Hamilton Rating Scale for Depression (HAMD-17)^24^ were eligible to participate in the study.

Patients were excluded from the study if they met the following criteria: (1) epilepsy or other unstable medical conditions; (2) a history of brain injury or surgery; (3) a history of manic, hypomanic, or mixed episodes; (4) investigational drug treatment within the previous 6 months; (5) a history of alcohol or drug abuse within the previous 12 months; (6) pregnancy or lactation; (7) currently cognitive behavioral therapy or other psychological therapies; (8) other brain stimulation therapies within the previous 12 months; or (9) a heart pacemaker.

### Run-in treatment and stabilization

All eligible patients were instructed to enter a 6-month run-in period during which they received antidepressant treatment in an open manner. The choice of antidepressants was based on the patients’ medication history and current condition, and the psychiatrists’ discretion. In general, paroxetine was prescribed for those who were not medicated at the time of entry, as it is a commonly used antidepressant in China^25,26^. The orally administered paroxetine dose in both the mono- and combination regimens was initiated at 10 mg/day and escalated to an optimal dose within 2 weeks based on the individual patient’s response, to a maximum dose of 40 mg/day. Patients who were already taking antidepressants, including paroxetine, continued their current regimen.

The severity of depressive symptoms was evaluated biweekly using the HAMD-17 during the run-in treatment period to determine whether patients had obtained full or partial remission, defined as a HAMD-17 score of ⩽7 and 8–14, respectively^27^. Stabilization had been achieved if the remission was maintained for at least 3 months over six consecutive assessment time points^27^. On a few occasions, the Young Mania Rating Scale (YMRS) was used to measure the severity of manic symptoms if a patient was experiencing a notable manic episode^28^. A manic switch was indicated by a YMRS score of 16 or higher^28^.

### Randomization and blinding

Patients who had achieved stable full or partial remission were randomly assigned to rTMS, antidepressants (ADP), or a combination of both (rTMS + ADP) in a ratio of roughly 1:1:1 using a random block scheme with complete and consecutive random numbers produced in advance. The group allocation was semi-blind, in which random codes were known by the rTMS therapists (Y.W., Z.H.W., Y.C.W.), but blind to other study personnel including the psychiatrists (M.C., Y.H.Z., R.Z.S., L.G., Y.T.Q., J.C.L., H.H.) who were responsible for pharmacological treatment, clinical assessors (H.N.W., X.X.W., R.G.Z.), and data collectors and analysts (H.N.W., X.X.W., Z.J.Z.). To maintain the blinding of the treatment condition, assessors, and psychiatrists communicated with patients separately and were instructed not to acquire information about their treatment conditions.

### Pharmacological maintenance treatment and adherence

The participants assigned to the ADP and rTMS + ADP groups continued their run-in antidepressant regimen as maintenance treatment for 12 months. Participants were required to attend biweekly or monthly appointments during the maintenance treatment. Adjustments to the dosage and the addition and termination of antidepressant medication were made at the discretion of the participant’s psychiatrist, on the basis of the individual’s response and tolerance. Concomitant use of other psychotropic medication was not generally allowed during the study period; however, as insomnia is a common comorbid symptom that would interfere with adherence if left untreated, the use of anti-insomnia medications was permitted for up to 7 cumulative days in each month.

Participants were required to keep a daily record of the number of tablets taken, lost, and remaining, which was checked by a psychiatrist on a biweekly or monthly basis. The adherence rate was calculated by dividing the number of tablets actually taken by the number the patient should have taken and multiplying by 100. Those whose adherence rate was less than 75% were defined as noncompliance cases.

### Clustered rTMS maintenance treatment and procedure

Participants who were assigned to the rTMS and rTMS + ADP groups received monthly clustered rTMS maintenance treatment, which involved 10 sessions over a 5-day period for the first 3 months and 5 sessions over a 3-day period thereafter. Those in the rTMS group were instructed to gradually withdraw their antidepressant drugs over 1–2 weeks, depending upon the pharmacokinetic properties of the antidepressant taken. The first clustered rTMS intervention started during antidepressant withdrawal.

The rTMS procedure was administered with a water-cooled, 100-mm, figure 8-shaped magnetic coil (MagPro R30, Dantec Medtronic, Denmark). The stimulation parameters have been adjusted to meet Chinese population as described previously^29^. Before the rTMS procedure, the individual’s resting motor threshold (RMT), defined as the stimulation intensity sufficient to evoke at least five motor evoked potentials (MEPs) in ten consecutive stimulations with 50 μV peak-to-peak amplitude, was determined on a daily basis. To obtain the MEPs, the coil was placed on the scalp corresponding to the left primary motor cortex and the stimulation intensity was gradually increased to elicit involuntary movement of the right abductor pollicis brevis. rTMS treatment was subsequently conducted by placing the coil on the left dorsolateral prefrontal cortex, defined as 5 cm anterior to the ipsilateral primary motor cortex used to determine the RMT, in the same parasagittal plane with the handle pointed backwards at a 45° angle. The stimulation parameters were 23 trains of 50 stimuli with a 35-s interval between trains at a frequency of 10 Hz. The intensity was set at 120% of RMT at first, but would be adjusted to 80% of RMT for few participants who could not tolerate. Each treatment involved a total of 1150 stimuli delivered over a 15-min period.

### Clinical assessments

The primary outcome measure was the time to first relapse, recurrence of depression, or switch to a manic episode during the 12-month study period. The severity of depressive symptoms was measured using the HAMD-17 and Clinical Global Impression-Severity (CGI-S) scale once a month. Relapse or recurrence of depression was defined as a HAMD-17 score of ⩾14, a CGI-S score of ⩾3 with an increase of at least two points, and meeting the DSM-IV criteria for a major depressive episode. The YMRS was used to measure the severity of manic symptoms if patients were experiencing a notable manic episode. A YMRS score of 16 or higher was classified as a manic switch. The secondary outcome was the relapse/recurrence rate at the endpoint. Safety and tolerability were evaluated using the Patient Rated Inventory of Side Effects (PRISE) to report side effects by patients themselves who may have experienced over the previous 7 days^30^.

To ensure the consistency and reliability of the clinical assessments over time, four training workshops were conducted pre-trial, once a year in 2013 and 2014, and at the completion of the whole study to test and re-test inter-rater (H.N.W., X.X.W., R.G.Z.) agreement. Substantial *κ* values^31^ of 0.65–0.87 were achieved. In most cases, the assessments for each individual were carried out by the same assessor throughout the run-in and maintenance treatment to minimize potential variations.

### Statistical analysis

The sample size estimation was based on relapse/recurrence rate. Based on four pilot controlled trials^10–13^ and four available case reports^17,19–21^, rTMS as mono- and additional therapy resulted in an average relapse rate of 42.4% compared to 68.4% with pharmacotherapy in long-term maintenance treatment. Therefore, a total of 276 participants (92 per group) were needed to detect a 26% difference in relapse/recurrence rates between the rTMS regimens and pharmacological treatment, with a power of 0.80 and alpha of 0.05, allowing for a dropout rate of 30%.

Efficacy was analyzed on the intention-to-treat population, defined as participants who completed the baseline and at least one evaluation after treatment. Survival analysis was performed to detect differences in the time to relapse or recurrence at 12 months among the three groups using a Cox regression proportional hazards model with adjustment for the number of previous depressive episodes, baseline depression severity, and antidepressants used. Those who discontinued and did not experience a relapse or recurrence during the 12 months of the study were treated as censored observations. Relapse/recurrence rates at the endpoint were examined using Chi-square (*χ* ^2^) test. Subgroup analyses were further conducted in relapsed/recurrent subjects to determine whether the treatment effects on relapse/recurrence rates were associated with the duration of the illness, previous depressive episodes, and remission status using a binary logistic regression model^31^. Continuous variables were analyzed using one-way analysis of variance (ANOVA) to evaluate between-group differences among the three groups. Categorical variables, including categorical baseline variables and incidence of adverse events were analyzed using Chi-square (*χ* ^2^) test. Statistical significance was defined as a two-tailed *P* < 0.05.

## Results

### Participant characteristics

**Fig. 1:**
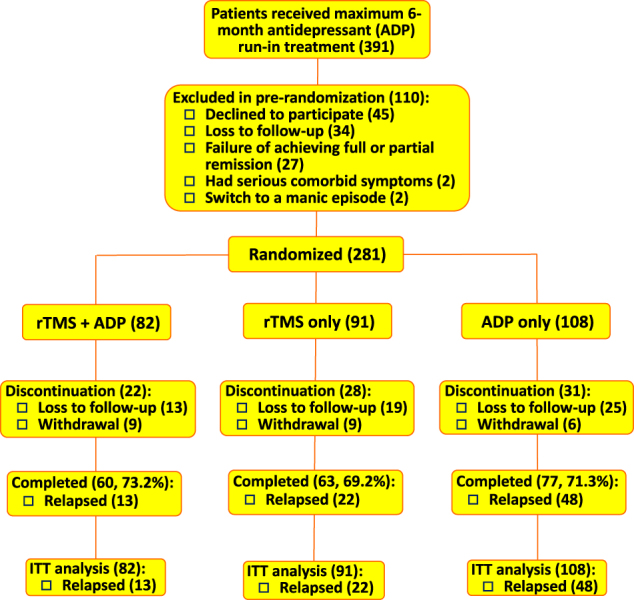
Flowchart of run-in and maintenance treatment of patients with a major depressive disorder. Intention-to-treatment (ITT) analysis was conducted. rTMS repetitive transcranial magnetic stimulation

Of the 391 patients who received run-in antidepressant treatment, 110 were excluded from pre-randomization (Fig. 1). Among them, 27 failed to achieve full or partial remission and 2 switched to a manic episode, respectively. The remaining 281 eligible participants were randomly assigned to the rTMS + ADP (*n* = 82), rTMS (*n* = 91), or ADP (*n* = 108) group. Overall, 71.2% (200/281) completed the full treatment and assessments as per the protocol. Eleven who failed to achieve the minimum adherence rate (<75%) were all noncompliant with antidepressants. Discontinuation rates were similar among the three groups. The profile of antidepressants run-in treatment was significantly different among the three groups (*χ* ^2^ = 19.759, d.f. = 8, *P* = 0.011). No statistically significant between-group differences were detected in the other baseline variables. A majority (62.6%, 176/281) of participants were in their first episode of major depression when they undertook the run-in treatment (Table 1).

**Table 1:** Baseline patient characteristics^a^

	rTMS + ADP (*n* = 82)	rTMS (*n* = 91)	ADP (*n* = 108)
Demographic characteristics			
Female, *n* (%)	60 (73.2)	66 (72.5)	85 (78.7)
Han ethnic origin, *n* (%)	82 (100)	91 (100)	108 (100)
Age, years			
Mean ± SD	42.3 ± 11.4	40.0 ± 11.5	40.9 ± 11.8
Range	19–63	19–64	18–64
Marital status			
Single, separated/divorced and widowed, *n* (%)	14 (17.1)	14 (15.4)	17 (15.7)
Married, *n* (%)	68 (82.9)	77 (84.6)	91(84.3)
Education			
Illiterate and elementary, *n* (%)	4 (4.8)	3 (3.3)	7 (6.5)
Middle, *n* (%)	24 (29.3)	24 (26.4)	36 (33.3)
High, *n* (%)	24 (29.3)	28 (30.7)	23 (21.3)
College and above, *n* (%)	30 (36.6)	36 (39.6)	42 (38.9)
Employment status			
Students, unemployed and retired, *n* (%)	34 (41.5)	36 (39.6)	36 (33.3)
Employed, *n* (%)	48 (58.5)	55 (60.4)	72 (66.7)
Clinical characteristics			
Family history of mental illness, *n* (%)	8 (9.7)	2 (2.2)	2 (1.9)
Duration of the illness, months			
Mean ± SD	14.5 ± 20.1	10.2 ± 11.3	14.2 ± 22.8
Range	1–120	1–60	1–120
No. of previous depressive episode			
0 (first episode), *n* (%)	51 (62.2)	52 (57.1)	73 (67.6)
1, *n* (%)	3 (3.7)	11 (12.1)	6 (5.6)
2–4, *n* (%)	22 (26.8)	27 (29.7)	26 (24.1)
≥5, *n* (%)	6 (7.3)	1 (1.1)	3 (2.8)3
Antidepressants taken in run-in treatment^b^			
Paroxetine	53 (64.6)	60 (65.9)	78 (72.2)
Venlafaxine	8 (9.8)	18 (19.8)	19 (17.6)
Escitalopram	7 (8.5)	6 (6.6)	9 (8.3)
Fluoxetine	12 (14.6)	4 (4.4)	2 (1.9)
Sertraline	2 (2.5)	3 (3.3)	0
Symptom severity at entry into run-in treatment			
HAMD-17, mean ± SD	22.9 ± 3.6	24.1 ± 3.6	24.0 ± 3.5
CGI-S, mean (SD)	4.5 ± 0.7	4.4 ± 0.7	4.5 ± 0.7
Symptom severity at entry into maintenance treatment			
HAMD-17, mean ± SD	6.5 ± 2.2	5.9 ± 3.0	5.8 ± 4.1
CGI-S, mean ± SD	1.3 ± 0.5	1.2 ± 0.4	1.3 ± 0.6
Remission			
Full (HAMD-17 ≤ 7), *n* (%)	55 (67.1)	73 (80.2)	85 (78.7)
Partial (HAMD-17 = 8–14), *n* (%)	27 (32.9)	18 (19.8)	23 (21.3)

^a^ *rTMS* repetitive transcranial magnetic stimulation, *ADP* antidepressants, *HAMD-17* the 17-item Hamilton Rating Scale for Depression, *CGS* Clinical Global Impression-Severity, *SD* standard deviation

^b^A significant difference was detected in antidepressants run-in treatment among the three groups (*χ* ^2^ = 19.759, d.f. = 8, *P* = 0.011)

The three most commonly used antidepressants in the run-in treatment were paroxetine (68.0%, 191/281), venlafaxine (16.0%, 45/281), and escitalopram (7.8%, 22/281). A significant between-group difference was observed in the run-in antidepressant profiles (*χ* ^2^ = 19.759, d.f. = 8, *P* = 0.011). Three quarters (75.8%, 213/281) had achieved full remission within 6 months of starting the run-in treatment (Table 1).

### Psychotropic medication profile of maintenance treatment

The psychotropic medications used in maintenance treatment are summarized in Table 2. The two most commonly used antidepressants in the rTMS + ADP and ADP groups were paroxetine (74.2%, 141/190) and venlafaxine (17.9%, 34/190). A significantly higher proportion took paroxetine in the ADP group than in the rTMS + ADP group (*χ* ^2^ = 4.524, d.f. = 1, *P* = 0.033). The proportion of participants co-medicated with benzodiazepines and non-benzodiazepines for insomnia did not significantly differ among the three groups.

**Table 2:** Psychotropic medications used in maintenance treatment^a^

	rTMS + ADP	rTMS	ADP	Statistical values		
	(*n* = 82)	(*n* = 91)	(*n* = 108)	*χ* ^2^ or *t*	d.f.	*P*
Antidepressants						
Paroxetine						
No. of patients (%)	54 (65.9)	—	87 (80.6)	4.524	1	0.033
Dose ( ± SD, mg/day)	20.1 ± 3.7	—	21.4 ± 4.5	1.857	139	0.065
Venlafaxine						
No. of patients (%)	15 (18.3)	—	19 (17.6)	0.004	1	0.947
Dose ( ± SD, mg/day)	132.1 ± 31.5	—	147.0 ± 16.5	2.018	32	0.085
Escitalopram						
No. of patients (%)	6 (7.7)	—	0	—	—	—
Dose ( ± SD, mg/day)	10.0 (0)	—	0	—	—	—
Fluoxetine						
No. of patients (%)	3 (3.8)	—	0	—	—	—
Dose ( ± SD, mg/day)	23.3 (5.8)	—	0	—	—	—
Sertraline						
No. of patients (%)	2 (2.6)	—	0	—	—	—
Dose ( ± SD, mg/day)	100.0 (0)	—	0	—	—	—
Anti-insomnia medications^b^						
Benzodiazepines, *n* (%)	50 (61.0)	64 (70.3)	65 (60.2)	2.570	2	0.277
Non-benzodiazepines, *n* (%)	11 (14.1)	19 (20.9)	15 (14.9)	1.771	2	0.412

^a^ *rTMS* repetitive transcranial magnetic stimulation, *ADP* antidepressants, *SD* standard deviation

^b^Benzodiazepines included diazepam, lorazepam, and alprazolam; non-benzodiazepines included zopiclone, zopiclone, and zolpidem

### Maintenance outcomes

**Fig. 2:**
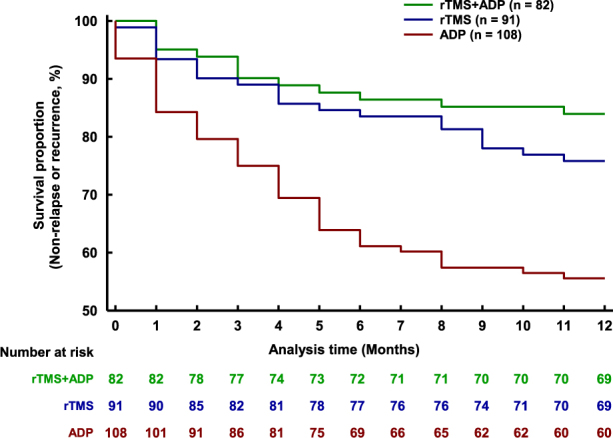
Cox regression proportional hazards model revealed that rTMS + ADP and rTMS treatment produced a significant reduction in the risk of relapse/recurrence compared with ADP, with the hazard ratios of 0.292 (*P* = 0.000) and 0.466 (*P* = 0.003), respectively. A hazard ratio of rTMS + ADP to rTMS of 0.637 did not reach statistical significance (*P* = 0.198). *rTMS* repetitive transcranial magnetic stimulation, *ADP* antidepressants

The primary outcome is illustrated in Fig. 2. The survival analysis revealed a significant difference in time to relapse/recurrence among the three groups. The rTMS + ADP and rTMS treatments produced a significant reduction in the risk of relapse/recurrence compared to ADP, with hazard ratio of 0.297 (*P* = 0.000) and 0.466 (*P* = 0.003), respectively. The hazard ratio of rTMS + ADP to rTMS of 0.637 did not reach statistical significance (*P* = 0.198).

Both rTMS-containing groups had strikingly lower relapse/recurrence rates than the ADP group (15.9% and 24.2% vs. 44.4%, *χ* ^2^ = 20.165, d.f. = 2, *P* < 0.001), with a difference of 28.5% between rTMS + ADP (*χ* ^2^ = 16.192, d.f. = 1, *P* = 0.001) and ADP and 20.2% between rTMS and ADP (*χ* ^2^ = 8.031, d.f. = 1, *P* = 0.005); but no significant difference was detected between rTMS + ADP and rTMS (*χ* ^2^ = 1.371, d.f. = 1, *P* = 0.242).

Five participants (three on rTMS + ADP and two on ADP) developed a manic episode during maintenance treatment. All of them were initially experiencing a first episode of major depression when they undertook the run-in treatment.

### Subgroup analysis of relapsed/recurrent subjects

To further determine the associations between demographic and clinical characteristics and relapse/recurrence, binary logistic regression analysis was performed on relapsed/recurrent subjects. A significant association was observed between the number of relapsed/recurrent subjects and the number of previous episodes (*χ* ^2^ = 6.054, d.f. = 2, *P* = 0.048). The relapse/recurrence rate of subjects with a first depressive episode was markedly lower in the of rTMS group than in the ADP group (*χ* ^2^ = 4.534, d.f. = 1, *P* = 0.033) (Table 3).

**Table 3:** Binary logistic regression model analysis of relapsed/recurrent subgroup, *n* (%)^a^

	rTMS + ADP	rTMS	ADP	Statistical values	
	(*n* = 13)	(*n* = 22)	(*n* = 48)	*χ* ^2^	*P* ^b^
Age				1.265	0.531
≤45 years	8 (61.5)	15 (68.2)	26 (54.2)	—	—
>45 years	5 (38.5)	7 (31.8)	22 (45.8)	—	—
Gender				0.717	0.699
Male	4 (30.8)	6 (27.3)	10 (20.8)	—	—
Female	9 (69.2)	16 (72.7)	38 (79.2)	—	—
Duration of the illness				1.030	0.597
≤12 months	8 (61.5)	17 (77.3)	33 (68.8)	—	—
>12 months	5 (38.5)	5 (22.7)	15 (31.2)	—	—
No. of previous episodes				6.054	0.048
None (first episode)	8 (61.5)	7 (31.8)	30 (62.5)	—	—
At least 1	5 (38.5)	15 (68.2)	18 (37.5)	—	—
Remission status				3.885	0.143
Partial	4 (30.8)	6 (27.3)	24 (50.0)	—	—
Full	9 (69.2)	16 (72.7)	24 (50.0)	—	—

^a^ *rTMS* repetitive transcranial magnetic stimulation, *ADP* antidepressants

^b^d.f. = 2

### Adverse events

The incidence of adverse events is summarized in Table 4. No patients discontinued treatment due to adverse events. The rTMS group exhibited a significantly higher incidence of headache, tremor, poor coordination, and difficulty in sleeping compared with the ADP group. The ADP group had a significantly higher incidence of dry mouth than the rTMS group. Patients in the combined treatment group reported significantly more excessive sweating compared with rTMS-treated patients, and were more likely to experience tinnitus, higher urine frequency, and loss of sexual desire than the ADP-treated patients.

**Table 4:** Incidence of adverse events, *n* (%)

	rTMS + ADP	rTMS	ADP	Statistical values	
	(*n* = 82)	(*n* = 91)	(*n* = 108)	*χ* ^2^	*P* ^b^
Diarrhea	5 (6.1)	6 (6.6)	8 (7.4)	0.133	0.936
Constipation	28 (34.1)	22 (24.2)	35 (32.4)	2.420	0.298
Dry mouth	43 (52.4)	28 (30.8)	66 (61.1)	18.827	<0.001
Nausea	3 (3.7)	7 (7.7)	8 (7.4)	1.464	0.481
Palpitations	11 (13.4)	10 (11.0)	9 (8.3)	1.276	0.528
Dizziness	8 (9.8)	14 (15.4)	8 (7.4)	3.398	0.183
Excessive sweating	32 (39.0)	16 (17.6)	32 (29.6)	9.854	0.007
Headache	6 (7.3)	13 (14.3)	2 (1.9)	11.046	0.004
Tremor	1 (1.2)	7 (7.7)	0	11.674	0.003
Poor coordination	0	7 (7.7)	0	14.989	<0.001
Blurred vision	21 (25.6)	23 (25.3)	15 (13.9)	5.345	0.069
Tinnitus	14 (17.1)	6 (6.6)	3 (2.8)	13.128	0.001
Urine frequency	17 (20.7)	6 (6.6)	7 (6.5)	12.278	0.002
Difficulty in sleeping	3 (3.7)	13 (14.3)	3 (2.8)	12.147	0.002
Sleeping too much	4 (4.9)	6 (6.6)	3 (2.8)	1.646	0.439
Loss of sexual desire	10 (12.2)	4 (4.4)	4 (3.7)	6.513	0.039
Trouble achieving orgasm	3 (3.7)	5 (5.5)	4 (3.7)	0.493	0.781
Anxiety	2 (2.4)	7 (7.7)	6 (5.6)	2.372	0.305
Difficulty in concentration	2 (2.4)	6 (6.6)	4 (3.7)	1.959	0.376
Tiredness	10 (12.2)	14 (15.4)	15 (13.9)	0.367	0.832
Decreased energy	4 (4.9)	8 (8.8)	7 (6.5)	1.069	0.586

^a^ *rTMS* repetitive transcranial magnetic stimulation, *ADP* antidepressants

^b^d.f. = 2

## Discussion

The present study showed that while the one-year relapse rate (44.4%) of antidepressant monotherapy was comparable to those (12–46%) of similar studies^33,34^ and the psychotropic medication profile was basically similar among the three groups during maintenance treatment, both rTMS-containing regimens significantly reduced the risk of relapse/recurrence and the relapse/recurrence rate by 20.2% and 28.5%, respectively, compared with antidepressant monotherapy. This result is in agreement with previous pilot controlled trials^10–13^ and case reports^17,19–21^. Furthermore, rTMS combined with antidepressants achieved remarkably better outcomes than rTMS alone in reducing the risk of relapse/recurrence and the relapse/recurrence rate by 8.3%, although the differences between the two groups did not reach statistical significance. All of these results demonstrate the superiority of rTMS, either alone or in combination with antidepressants, over antidepressant monotherapy in the long-term prevention of depressive relapse and recurrence.

Similar to most studies of rTMS treatment of depressive disorders^7^, the present study also used high-frequency (10 Hz) rTMS on the left dorsolateral prefrontal cortex. This rTMS paradigm has been recommended as a standard protocol for the treatment of depressive disorders^5^. In addition to facilitating synaptic plasticity in the brain, high-frequency rTMS (>5 Hz) exerts multiple non-synaptic and neurochemical effects^9^, and modulates various neurotransmitters^35–38^. The rTMS treatment clearly has broader neuromodulatory effects than the antidepressants, and these modulatory effects are enhanced as the number of treatment sessions increases over the long-term^9^. This could explain, at least in part, the superior efficacy of rTMS as a monotherapy over antidepressants observed in this study. High-frequency rTMS combined with selective noradrenaline reuptake inhibitors produces a synergistic effect on motor functions in healthy humans^39^. This is consistent with the finding of the present study, showing the superiority of rTMS combined with antidepressants over rTMS alone in preventing depressive relapse. It seems that the addition of rTMS could augment antidepressant efficacy, probably via additive or synergistic interactions. On the other hand, although the dose of TMS used in this study was relatively low on stimuli pulse number and intensity in few participants compared to previous studies^5,7^, the equivalent efficacy was still achieved. It appears that depressed patients from different ethnic groups may have different tolerance but have similar response to rTMS.

Unlike most “usual” rTMS maintenance regimens used in previous studies, where one or two sessions were evenly delivered at weekly, fortnightly, or monthly intervals^10–13,40^, our study used a clustered rTMS maintenance approach, in which more frequent and concentrated sessions were administered within a short period (for example, 3–5 days) on a monthly basis. One observational study has shown the potential of clustered maintenance rTMS in delaying the occurrence of depressive relapse^19^. A notable advantage of clustered rTMS is that it may promote adherence to maintenance treatment compared with pharmacotherapy and the “usual” rTMS regimens. Indeed, we found that 11 participants who did not achieve the minimum adherence rate (<75%) failed to comply with the antidepressants, but not the rTMS treatment. Noncompliance and discontinuation are a common issue in antidepressant long-term maintenance treatment and are heavily associated with a high rate of relapse and recurrence^41,42^. Therefore, the superior maintenance outcomes obtained in the rTMS-containing regimens may be partly due to good adherence with the clustered maintenance rTMS.

A characteristic of the participants in the present study is that approximately two thirds of them were experiencing their first episode of major depression at intake. This led to concern over whether rTMS could increase the risk of manic switch, as it is somewhat difficult to differentiate between unipolar and bipolar depression in the first-episode depressed population. There have been several case reports of switching to acute hypomania and mania during and following rTMS treatment in patients with a major depressive episode^43–47^. In the current study, however, three participants in the combination maintenance and two in the antidepressant group, but none in the rTMS group, experienced a manic episode during maintenance treatment. Two further patients developed acute mania during the antidepressant run-in treatment. All of them were experiencing their first episode of major depression at entry into the run-in treatment. Although we could not exclude that these manic switches probably represent a first episode of mania in the clinical course of bipolar disorder, our results suggest that long-term maintenance with antidepressants, rather than rTMS, is more closely associated with a higher risk of manic switch in first-episode depressed patients. The subgroup analysis of relapsed/recurrent subjects further revealed that rTMS-maintained patients with a first depressive episode had a much lower relapse/recurrence rate than those taking antidepressants. Altogether with the findings of previous studies that showed that rTMS accelerated antidepressant response in first-episode young depressive patients^29,48^, it seems that rTMS may have specific benefits for patients with a first-episode depressive disorder in enhancing rapid antidepressant effects and preventing the reoccurrence of depressive episodes.

Consistent with previous studies^32^, this study also revealed that long-term maintenance rTMS was well tolerated, without related adverse events leading to discontinuation of treatment. However, patients on rTMS alone experienced more neurological side effects, including headache, tremor, and poor coordination as well as difficulty in sleeping, compared with pharmacotherapy. Similar side effects, such as seizures, transient headaches, and dizziness, have also been observed in rTMS short-term treatment^49,50^. Patients on the combined treatment also had a higher incidence of excessive sweating, tinnitus, urine frequency, and loss of sexual desire. These data suggest that rTMS as a long-term mono- and additional therapy may increase the risk of nervous system side effects, which should be considered with caution when rTMS serves as long-term preventive intervention.

Several limitations of this study should be noted. First, sham rTMS was not included as an inactive control and patients were aware of their assignment. This was necessary because one objective of our study was to evaluate participants’ acceptance and adherence with clustered rTMS long-term maintenance in an open manner. Despite this, the assessors and most of the other investigators were blind to the patients’ treatment. Such a blinded outcome assessment design can minimize bias in open-label trials^51^. Second, it is unclear if clustered rTMS could be superior to those “usual” rTMS regimens in achieving maintenance efficacy, safety, and tolerability, although this study, together with a previous study^19^, confirmed the value of this novel maintenance rTMS in preventing depressive relapse. A further comparison between “usual” and clustered rTMS regimens may be warranted. Third, although rTMS monotherapy achieved the remarkable efficacy in the participants who were required to gradually discontinue antidepressants when the maintenance treatment was initiated, this may place them at significant risk of relapse. Closer monitoring should be provided for these participants. Finally, participants of this study were those who had achieved partial or full remission during run-in treatment with antidepressants, rather than with rTMS. This was based on the fact that pharmacotherapy is still the mainstay in the management of depressive disorders and rTMS is mainly used as additional or add-on therapy in treatment-resistant subjects^4,8^. Whether rTMS as run-in and maintenance treatment could achieve similar outcomes in treatment-resistant depressed populations needs further evaluation.

Collectively, our study demonstrates that rTMS as a mono- and additional maintenance therapy is superior to antidepressants in preventing depressive relapse, particularly in first-episode depressed patients, without increasing the risk of manic switch, but potentially increasing the risk of certain side effects. Our findings indicate that rTMS can be considered as an effective preventive maintenance treatment for depressive relapse.
